# *Cataphryxus zapoteca* sp. nov. (Isopoda, Bopyridae) and new hosts and records of bopyrid isopods from the Mexican Eastern Pacific

**DOI:** 10.1007/s11230-023-10118-z

**Published:** 2023-10-17

**Authors:** Jesús Romero-Rodríguez, Fernando Álvarez

**Affiliations:** Colección Nacional de Crustáceos, Instituto de Biología, Universidad Nacional Autónoma de México (UNAM), Apartado Postal 70–153, 04510 Mexico City, Mexico

## Abstract

Based on the examination of diverse crustacean taxa collected along the Mexican Pacific and deposited in the Colección Nacional de Crustáceos of the Instituto de Biología, UNAM, six species of bopyrid isopods were detected. New hosts and localities are reported for *Munidion pleuroncodis* Markham, 1975, *Probopyrus pacificensis* Román-Contreras, 1993, *Probopyrus markhami* Román-Contreras, 1996, *Progebiophilus bruscai* Salazar-Vallejo & Leija-Tristán, 1990 and *Schizobopyrina striata* (Nierstrasz & Brender à Brandis, 1929). *Cataphryxus zapoteca* **sp. nov.**, is described as abdominal parasite of the shrimp *Lysmata galapagensis* Schmitt; this bopyrid is the second species described in the genus *Cataphryxus* Shiino, 1936 and the first registered on the American continent. Taxonomic characters, distribution and some reproductive data for five of the six species examined are provided in order to update the knowledge of this parasite group in this Eastern Pacific region.

## Introduction

Bopyrid isopods are obligate parasites of other crustaceans and to complete their life-cycle, they need two different groups of crustacean hosts: copepods as intermediate hosts and decapods, as definitive hosts (Cericola & Williams, 2015). Bopyrid females have large and asymmetric bodies, so much so that in some species they are barely recognizable as isopods; in contrast, males are small and similar in form to free-living isopods (Román-Contreras, 2008; Williams & Boyko, 2012). These parasites can negatively impact the host biology and ecology by causing metabolic, behavioral, physiological and reproductive alterations (Román-Contreras, 2008).

According to Markham (1992), the first two bopyrid isopods recorded from the eastern Pacific coast were *Argeia pugettensis* Dana, 1853 parasitizing *Crangon munita* (Dana), cited as *C. munitus*, in Puget Sound (Dana, 1853) and *Phyllodurus abdominalis* Stimpson, 1857 parasitizing a “common *Gebia*” at Puget Sound and Tomales Bay, in the northwest coast of US (Stimpson, 1857); subsequently in the early 1900s Richardson (1905) recognized 13 bopyrid species distributed along the Pacific coast, of which 11 remain accepted today. Markham (1992) recognized 29 described and 10 undescribed bopyrid species parasitizing 86 species of host decapods along the Eastern Pacific coast, later the number found in this region was estimated at 37, which represents only 22.3 % of the 166 species reported for the whole Western Pacific (Williams & Boyko, 2012). The underrepresentation of bopyrids along in the Eastern Pacific seems to be product of an inadequate sampling as the number of potential hosts is probably not a limiting factor (Markham, 1992).

In the Mexican Pacific, three deep sea bopyrids were the first reported: *Bathygyge grandis* Hansen, 1897 parasitizing *Glyphocrangon spinulosa* Faxon off the coast of Nayarit at a depth of ~1236 m, *Pseudione galacanthae* Hansen, 1897 parasitizing *Galacantha diomedeae* Faxon in the Gulf of California at a depth of ~1571 m, and *Parargeia ornata* Hansen, 1897 parasitizing *Metacrangon procax* (Faxon), cited as *Sclerocrangon procax*, off the coast of Guerrero at a depth of ~1207 m (Hansen, 1897). There were no additional records of bopyrids for almost 80 years (Román-Contreras, 2008), until the reviews of the genera *Stegophryxus* Thompson, 1902 and *Munidion* Hansen, 1897 were published (Markham, 1974, 1975). Currently, based on checklists included in some ecological reports (Campos & Campos, 1989a; Salazar-Vallejo & Leija-Tristán, 1990), bibliographical compilations (Román-Contreras, 2008) and recent reports of these parasites (Romero-Rodríguez & Álvarez, 2019), bopyrid diversity in the region comprises 19 species found parasitizing a wide diversity of crustaceans.

During examination of biological material deposited in the Colección Nacional de Crustáceos (CNCR), housed at the Instituto de Biología of the Universidad Nacional Autónoma de México, parasitized crustaceans of diverse taxa were detected thus the aim of this study is to report the taxonomic identity, host selection, distribution and reproductive data on the bopyrid species examined in order to update the knowledge of this parasite group along the Mexican Pacific.

## Material and methods

The abdomen and carapace of diverse taxa of crustaceans formerly collected along the Mexican Pacific and deposited in the CNCR were examined for bopyrid isopods. The size, considered as carapace length (CL) and sex for each host were registered. The bopyrids were gently removed from their hosts in order to recognize their specific identities and to record the size of each parasite, that was considered as total length (TL) and measured from the anterior margin of the first pereomere of the longer side to the posterior margin of the pleon (Romero-Rodríguez & Álvarez, 2020). For each bopyrid ovigerous female fecundity was estimated by direct counting of embryos, except in brood pouches with large numbers of embryos in which a subsample was counted directly and the number of embryos was calculated by extrapolation. Embryos were classified as: egg, embryo I, embryo II and epicaridium larvae following Romero-Rodríguez and Román-Contreras (2013). Ten embryos were randomly selected from each brood pouch, their width (d^1^) and length (d^2^) were measured and their volume (V) calculated with the formula V = π(d^1^)^2^ * (d^2^)/6 (Romero-Rodríguez & Román-Contreras, 2013; Cericola & Williams, 2015). Measurements were made to the nearest 0.1 mm using an ocular micrometer attached to a compound microscope. Drawings made with a camera lucida were used to construct figures using Adobe Illustrator. Digital photographs of bopyrids were taken with a Leica DFC490 camera mounted on a Leica Z16APOA stereomicroscope provided with the Leica Application Suite version 4.3.0.

## Systematics

Suborder Epicaridea Latreille, 1825

Family Bopyridae Rafinesque, 1815

Subfamily Bopyrinae Rafinesque, 1815

Genus *Probopyrus* Giard and Bonnier, 1888

***Probopyrus pacificensis*** Román-Contreras, 1993

**Fig. 1:**
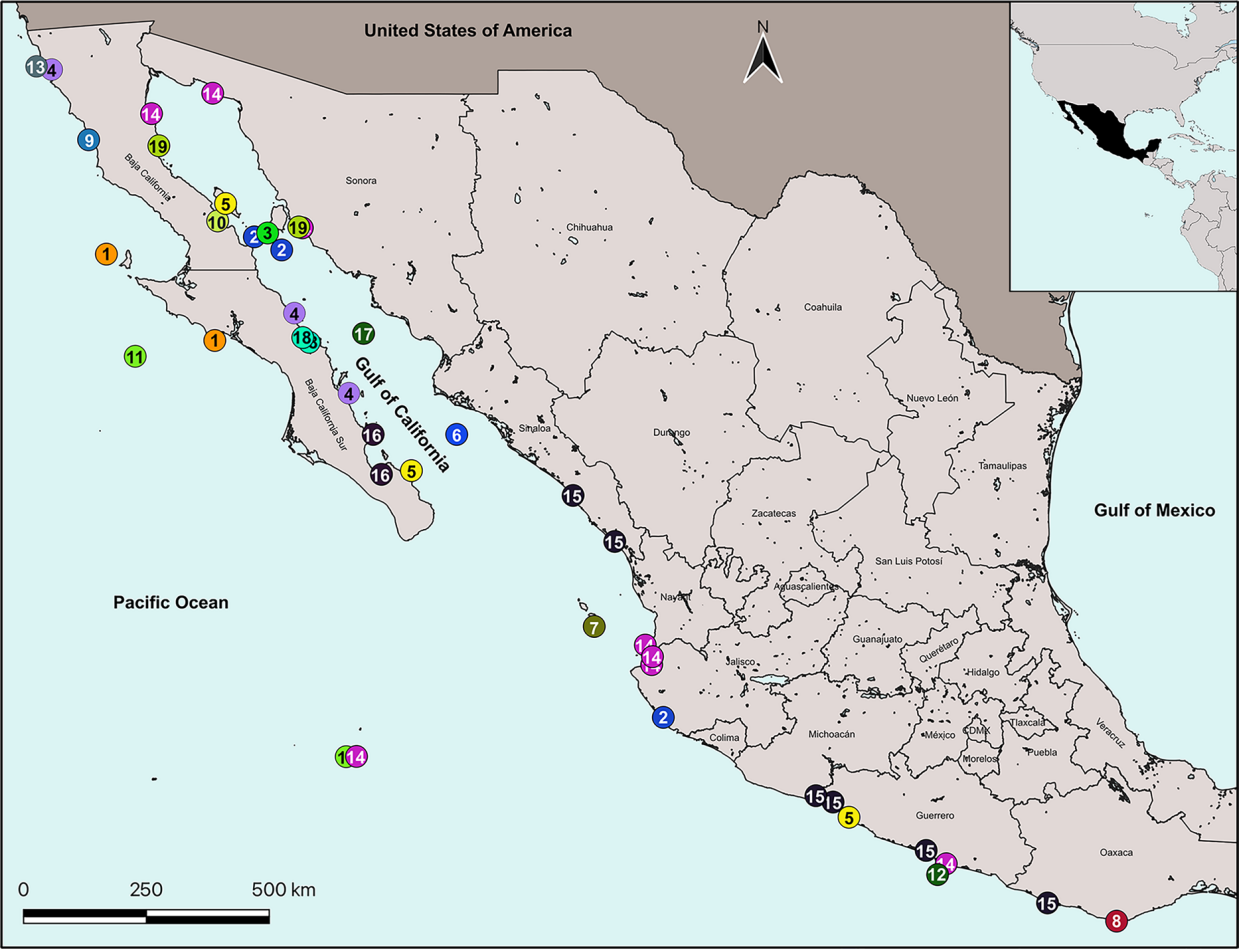
Bopyrid species distribution along the Pacific coast of Mexico. **1.** *Anathelges hyphalus* (Markham, 1974). **2.** *Aporobopyrus bourdonis* Markham, 2008. **3.** *Aporobopyrus curtatus* (Richardson, 1904). **4.** *Aporobopyrus muguensis* Shiino, 1964. **5.** *Aporobopyrus trilobatus* (Nierstrasz & Brender à Brandis, 1925). **6.** *Bathione magnafolia* Román-Contreras & Boyko, 2007. **7.** *Bathygige grandis* Hansen, 1897. **8.** *Cataphryxus zapoteca* **sp. nov. 9.** *Leidya infelix* Markham, 2002. **10.** *Ione cornuta* Spence Bate, 1863. **11.** *Munidion pleuroncodis* Markham, 1975. **12.** *Parargeia ornata* Hanse, 1897. **13.** *Phyllodurus abdominalis* Stimpson, 1857. **14.** *Probopyrus pacificensis* Román-Contreras, 1993. **15.** *Probopyrus markhami* Román-Contreras, 1996. **16.** *Progebiophilus bruscai* Salazar-Vallejo & Leija-Tristán, 1990. **17.** *Pseudione galacanthae* Hanse, 1897. **18.** *Schizobopyrina bruscai* Campos & Campos, 1990. **19.** *Schizobopyrina striata* (Nierstrasz & Brender à Brandis, 1929)

**Fig. 2:**
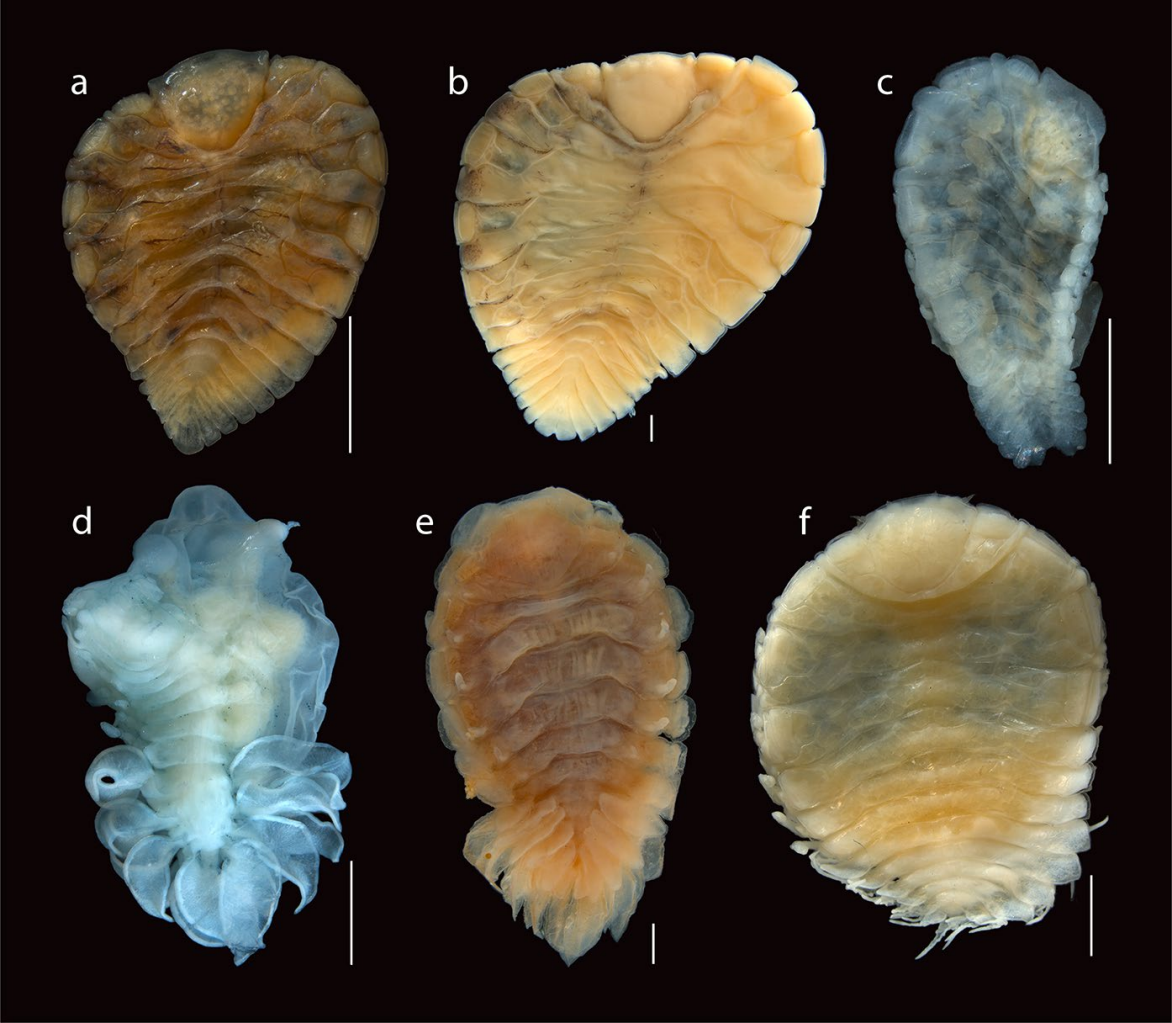
Dorsal view of adult bopyrid isopod females that parasitize crustaceans on the Mexican Pacific coasts. **a.** *Probopyrus pacificensis* CNCR-36921-B. **b.** *Probopyrus markhami* CNCR-36923-B. **c.** *Schizobopyrina striata* CNCR-36924. **d.** *Cataphryxus zapoteca* **sp. nov.** CNCR-36925. **e.** *Munidion pleuroncodis* CNCR-36926-D. **f.** *Progebiophilus bruscai* CNCR-36927. Scale bars = 1.0 mm

**Fig. 3:**
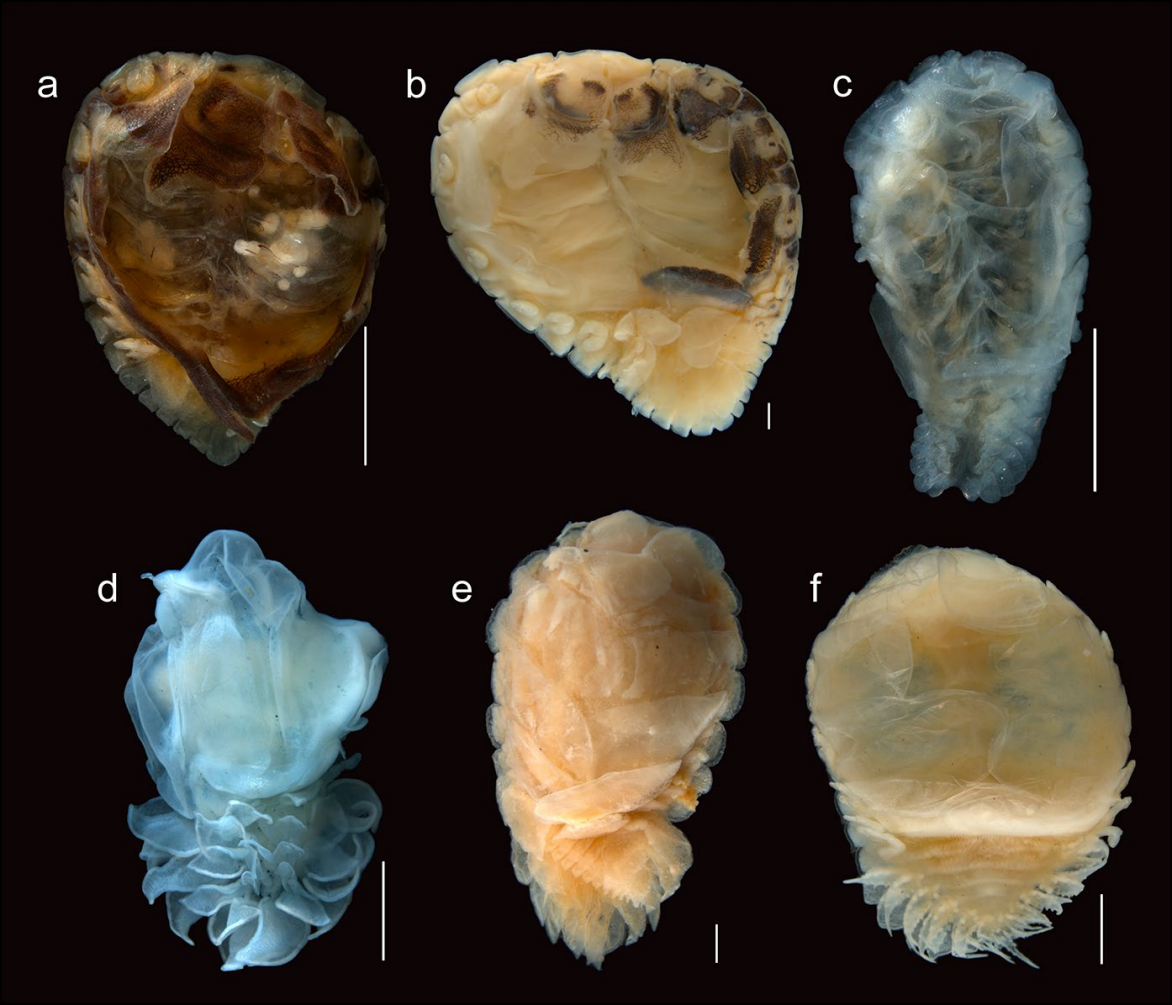
Ventral view of adult bopyrid isopod females that parasitize crustaceans on the Mexican Pacific coasts. **a.** *Probopyrus pacificensis* CNCR-36921-C. **b.** *Probopyrus markhami* CNCR-36923-B. **c.** *Schizobopyrina striata* CNCR-36924. **d.** *Cataphryxus zapoteca* **sp. nov.** CNCR-36925. **e.** *Munidion pleuroncodis* CNCR-36926-D. **f.** *Progebiophilus bruscai* CNCR-36927. Scale bars = 1.0 mm

**Fig. 4:**
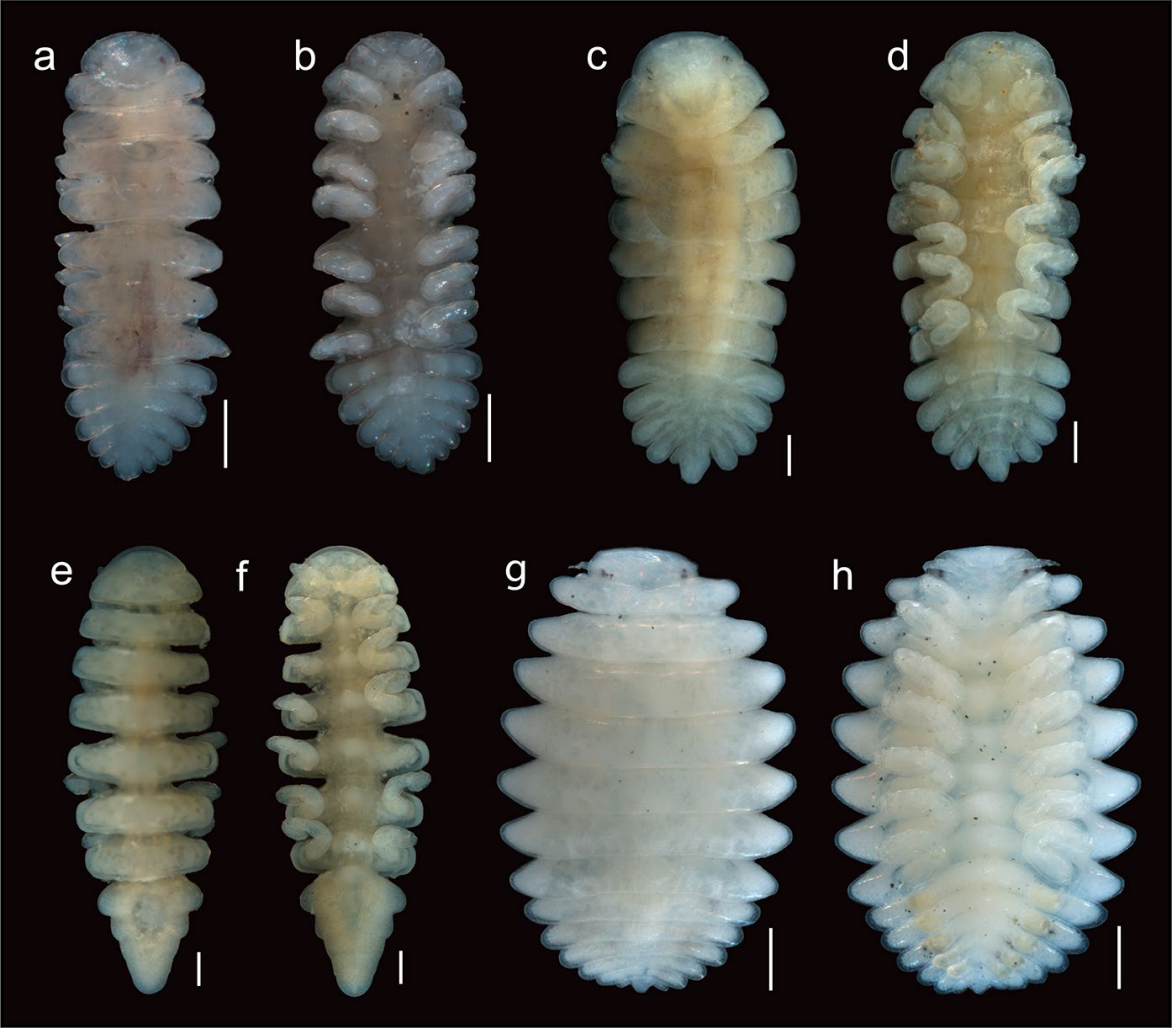
Adult bopyrid isopod males that parasitize crustaceans on the Mexican Pacific coasts. **a.** *Probopyrus pacificensis* CNCR-36922-B, dorsal view. **b.** same, ventral view. **c.** *Probopyrus markhami* CNCR-36923-B, dorsal view. **d.** same, ventral view. **e.** *Munidion pleuroncodis* CNCR-36926-F, dorsal view. **f.** same, ventral view. **g.** *Progebiophilus bruscai* CNCR-36927, dorsal view. **h.** same, ventral view. Scale bars = 0.2 mm

**Fig. 5:**
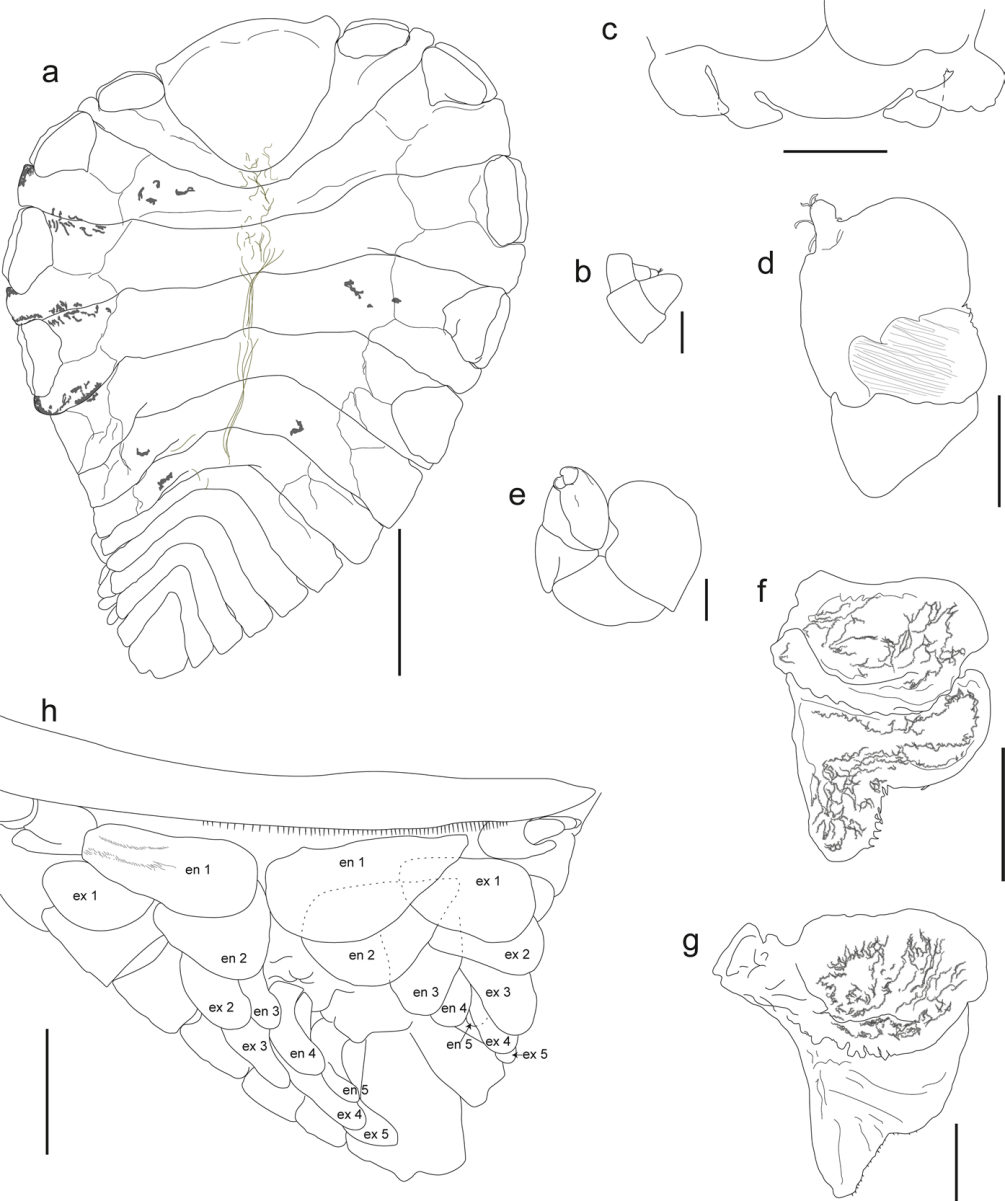
*Probopyrus pacificensis* CNCR-36922-A, except **f**, CNCR-36921-A. **a,** Ovigerous female, dorsal view. **b.** Antennule and antennae. **c.** Barbula. **d.** Maxilliped. **e.** Right pereopod 7. **f.** Right oostegite 1, internal view. **g.** Right oostegite 1, internal view. **h.** Pleon, ventral view. Scale bars: **a** = 1.0 mm. **b** and **e** = 0.1. **c**, **d** and **f**-**h** = 0.5 mm. Numbers indicates the corresponding pleopod. Abbreviations: **en**, endopod; **ex**, exopod

Figs. 1, 2A, 3A, 4A-B, 5; Table 1

**Table 1:** Reproductive traits of the examined bopyrid isopods parasitizing crustaceans in the Mexican Pacific coast. *Females with evident loss of embryos. ^a^estimated by indirect method

Species	n	Embryo stage	Fecundity ± D.S.	Mean embryo size ± D.S.		
				Length (mm)	Width (mm)	Volume (mm^3^)
*Munidion pleuroncodis*	7	I	11134.57 ± 4360.25	0.166 ± 0.013	0.158 ± 0.012	0.0022 ± 0.0005
	1	II	6413	0.169 ± 0.013	0.153 ± 0.009	0.0021 ± 0.0004
	1	epicaridium	7431	0.232 ± 0.12	0.144 ± 0.011	–
*Probopyrus pacificensis*	4	I	2687.67 ± 401.58	0.163 ± 0.12	0.147 ± 0.013	0.0019 ± 0.0004
	1	II	823	0.182 ± 0.009	0.147 ± 0.010	0.0021 ± 0.0004
*Probopyrus markhami*	1	I	<100*	0.179 ± 0.014	0.170 ± 0.016	0.0028 ± 0.0007
	1	epicaridium	27038.5^a^	0.284 ± 0.021	0.149 ± 0.011	–
*Progebiophilus bruscai*	1	II	4416	0.176 ± 0.009	0.147 ± 0.002	0.0020 ± 0.0002
*Schizobopyrina striata*	1	I	< 30*	0.120 ± 0.007	0.115 ± 0.008	0.0008 ± 0.0002

Bopyrids.– Holthuis, 1954: 6, 7 [Zunzal and Conchalió Rivers, El Salvador; parasitizing *Macrobrachium tenellum* (Smith)].

*Probopyrus* sp.– Román-Contreras, 1979: 157 [Laguna Tres Palos, Guerrero, Mexico; same host].– Román-Contreras, 1983: 361.

*Probopyrus pandalicola*.– Guzmán & Román-Contreras, 1983: 345–357 [Guerrero and Michoacán, Mexico; same host].– Román-Contreras, 1991: 112, 115, 119 [Laguna de Coyuca, Guerrero, Mexico; same host]. [not *Probopyrus pandalicola* (Packard, 1879)].

*Probopyrus pacificensis* Román-Contreras, 1993: 689–697, figs. 1–2 [type locallity: Laguna Tres Palos, Guerrero, Mexico, same host].

*Probopyrus pacificensis*.– Román-Contreras, 1996: 208.– Rodríguez-Almaraz et al., 2000: 860 [Puerto Peñasco, Sonora, Mexico, parasitizing *Palaemon ritteri* Holmes].– Dreyer & Wägele, 2001: 159, figs. 1, 3, 4, 6–9, Tables 1, 3.– Espinosa-Pérez & Hendrickx, 2001: 50.– Román-Contreras & Bourdon, 2001: 918, 922, 927, Tables 1, 2.– Román-Contreras & Soto, 2002: 285.– Román-Contreras, 2004: 153–156, 161, fig. 3, Tables 1, 2.– Román-Contreras & Romero-Rodríguez, 2005: 86, 87.– Román-Contreras, 2008: 95, 96, 98, 102, figs. 1, 2, 4, 7, Table 1.– Ocaña-Luna et al., 2009: 259–261, fig. 1, Table 1 [San Francisco creek, Nayarit and Palo María creek, Jalisco, Mexico; parasitizing *M. tenellum*].– Saito et al., 2010: Table 1.– Boyko et al., 2013: fig.1.– Romero-Rodríguez & Román-Contreras, 2013: 645.– Sivasubramanian et al., 2015: fig. 2.– Vargas-Ceballos et al., 2016: 34–44, fig. 2 [Ameca river, Jalisco, Mexico; same host].– Hendrickx et al. 2019: Table 7.– An et al., 2020: fig. 2.– Bortolini et al., 2021: table 2.– Aguilar-Perera, 2022: 114, Table 1.– García-Madrigal et al., 2022: Table 1.– Kato et al., 2022: fig. 11.– Wu et al., 2022: fig. 1.

Material examined.– 2 ovigerous females (3.92 ± 0.40 mm TL), 1 adult female (3.33 mm TL), 1 cryptoniscus larva (0.84 mm TL) and 2 males (0.99 ± 0.01 mm TL) (CNCR-36921) parasitizing 2 females (4.30 ± 0.28 mm LC) and 1 male (4.15 mm LC) of *Palaemon hiltoni* (Schmitt) (CNCR-19680); M. Martínez-Mayén det. host; Estero Santa Cruz, Bahía de Kino, Sonora, Mexico (28°47'55"N 111°54'58"W); Villalobos et al. colls.; 28 March 1983. 3 ovigerous females (5.71 ± 0.44 mm TL) and 3 males (1.64 ± 0.09 mm TL) (CNCR-36922) parasitizing 2 females (6.57 ± 0.14 mm LC) and 1 male (6.0 mm LC) of *Palaemon ritteri* (CNCR-34120); M. Martínez-Mayén det. host; Isla Socorro, Colima, Mexico (18°46'57"N 110°58'05"W); 25 January 2008.

Distribution.– *Probopyrus pacificensis* has been mainly collected on the branchial chamber of *Macrobrachium tenellum* that inhabits coastal lagoons and associated rivers and ponds from southern Nayarit to Guerrero, Mexico (Román-Contreras, 1993; Ocaña-Luna et al., 2009). Rodríguez-Almaraz et al. (2000) extended the northern range of this bopyrid to Puerto Peñasco, Sonora, parasitizing *Palaemon ritteri* (Fig. 1) and its presence was suggested in the Zunzal and Conchalió Rivers, El Salvador, parasitizing *M. tenellum* and on the Atlantic coast of Panama parasitizing *M. acanthurus* (Román-Contreras, 2004). Here we report for the first time to *P. pacificensis* from Socorro Island, Colima, parasitizing *P. ritteri* (Fig. 1), and *Palaemon hiltoni* is reported by first time as host of *P. pacificensis*.

Remarks.– The material examined conforms well with the characteristics proposed for *P. pacificensis* (Figs. 2A, 3A, 4A-B, 5A) by Román-Contreras (1993): antennule and antenna of three and two segments, respectively (Fig. 5B), barbula with two lateral projection on each side, inner smaller and slenderer than external one (Fig. 5C), palp of the maxilliped ovoid with 6 to 10 setae (Fig. 5D), all pereopod bases with conspicuous and rounded carina (Figs. 5E), as well as long pleopods that reach or protrude from the lateral margin of the pleon (Figs. 5A). Two variations of the species noted by Román-Contreras (1993) also were observed: the internal ridge of first oostegite ranged from slightly sinuous (n = 2) (Fig. 5F) to digitate (n = 2) (Fig. 5G), and the notch in the pleotelson ranged from absent (n = 1) (Fig. 2A) or slight (n = 1) (Fig. 5H) to conspicuous (n = 4) (Fig. 5A). However, the following differences from the original description were noted: females with scarce dorsal pigmentation (Figs. 2A), especially in those parasitizing *P. ritteri* (Fig. 5A); pereomeres 5–7 of both sides of the body with lateral margins square and close together to each other (Figs. 2A, 5A), and the middle margin of the barrbula elevated forward but not acute (Fig. 5C). Pleopods were not described in detail but those of specimens examined were of different shape and size, with pairs 1 and 2 foliaceous with the endopod wider than the exopod but with pairs 3–5 oblong in outline with both rami of similar width; the length of both rami progressively decreasing posteriorly but in pairs 1 and 2 the endopod is similar or longer than the exopod while in pairs 3–5 the endopod is progressively smaller than the exopod (Fig. 5H).

*Probopyrus pacificensis* and *P. pandalicola* are quite similar but they have been recognized as distinct genetically (Dreyer & Wägele, 2001; An et al., 2020; Kato et al., 2022) and morphologically can be distinguished as *P. pandalicola* has both the antennule and antenna three-segmented, two obtuse points in the middle margin of the barbula, maxilliped with subtrapezoidal palp, basis of first pereopod with reduced or absent carina and first pair of pleopods large and nearly concealing all others (see Markham, 1985; Román-Contreras, 2008). Based on these morphological differences and host selection, Román-Contreras (2004) questioned the presence of *P. pandalicola* in the eastern Pacific, which was previously reported parasitizing *Palaemon ritteri* in the northeast coast of Baja California, Mexico (Campos & Campos, 1989a) and *Palaemon hiltoni* in the Gulf of Nicoya, Costa Rica (Jiménez & Vargas, 1990); but because both reports precede the description of *P. pacificensis* it is probable that they actually correspond to this latter species, especially those parasitizing *P. hiltoni* as Jiménez & Vargas (1990) pointed out differences and doubted the identification of their specimens as *P. pandalicola*. Román-Contreras (2004) reported *P. ritteri* parasitized by a *Probopyrus* sp. at two islands of the Gulf of California, Mexico, and described some characters of these specimens that match those of *P. pacificensis* (see Román-Contreras, 2004, Table 2). The above allow us to suggest that the distribution of *P. pandalicola* is restricted to the west Atlantic coasts and that of *P. pacificensis* to the eastern Pacific region.

The average fecundity and embryo size for two stages of development of *P. pacificensis* are in table 1. Overall, fecundity of *P. pacificensis* agrees with the range of 350–11850 embryos reported for *P. pandalicola* (Beck, 1980) but by developmental stages it was lower than the mean brood size estimated by Beck (1980) for egg (3920 embryos) and embryo I stages (4154.2 embryos). The embryo size of both developmental stages of *P. pacificensis* are similar to those reported for egg (length and width of 0.15 mm) and embryo I (length of 0.18 – 0.20 mm, width 0.15 – 0.18 mm) of *P. pandalicola* (Beck, 1980).

***Probopyrus markhami*** Román-Contreras, 1996

**Fig. 6:**
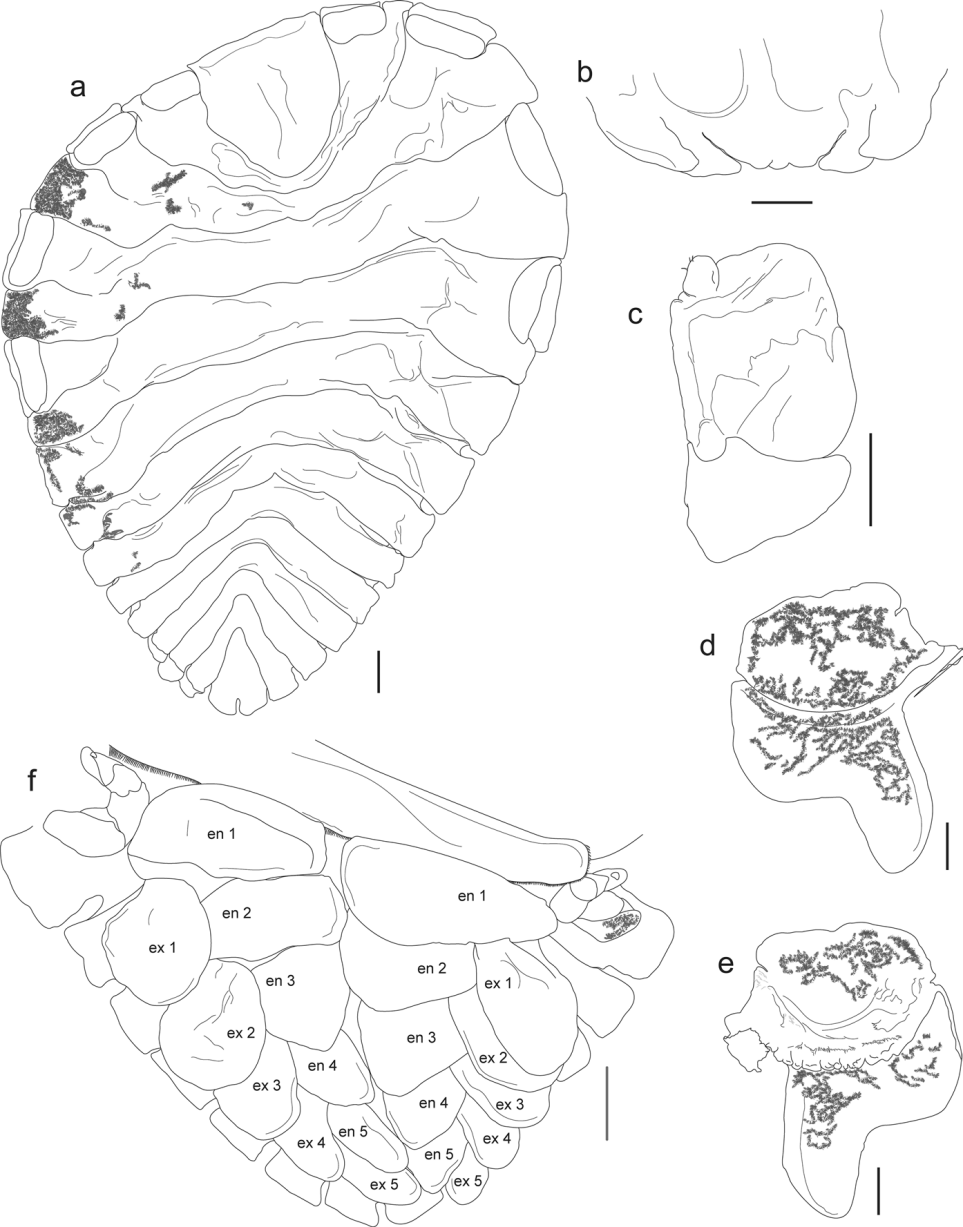
*Probopyrus markhami* CNCR-36923-A. **a.** Ovigerous female, dorsal view. **b.** Barbula. **c.** Maxilliped. **d.** Left oostegite 1, external view. **e.** same, internal view. **f.** Pleon, ventral view. Scale bars = 1.0 mm. Numbers indicates the corresponding pleopods. Abbreviations: **en**, endopod; **ex**, exopod

Figs. 1, 2B, 3B, 4C-D, 6; Table 1

*Probopyrum* (sic).– García-Bojórquez, 1983: 7 [parasitizing *Macrobrachium americanum* Bate].

*Probopyrus markhami* Román-Contreras, 1996: 204–209, figs. 1–15 [type locallity: La Unión River, Guerrero, Mexico, same host].

*Probopyrus markhami*.– Espinosa-Pérez & Hendrickx, 2001: 50.– Román-Contreras & Bourdon, 2001: 918, 922, 927, Tables 1, 2.– Román-Contreras, 2004: 153, 156, 161, fig. 3, Tables 1, 2.– Brusca et al., 2005: 137.– Román-Contreras, 2008: 98, Table 1.– Ocaña-Luna et al., 2009: 259, 260.– Saito et al., 2010: Table 1.– Vargas-Ceballos et al., 2016: 40.– Hendrickx et al. 2019: Table 7.– García-Madrigal et al., 2022: Table 1.

Material examined.– 2 ovigerous females (18.01 ± 1.05 mm TL) and 2 males (3.07 ± 0.46 mm TL) (CNCR-36923) parasitizing 1 female (23.5 mm CL) and 1 male (27.0 mm CL) of *Macrobrachium americanum* (CNCR-25470). F. Álvarez det. host; Río Verde, Oaxaca, Mexico (16°00'48"N 97°47'28"W); F. Álvarez and J. L. Villalobos colls.; 16 December 2008.

Distribution.– Román-Contreras (2004) considered *Probopyrus markhami* as endemic to Mexico because its known distribution range was from Piaxtla River, Sinaloa, to Coyuca River, Guerrero (Román-Contreras, 1996); here we extend its southern range to Río Verde, Oaxaca (Fig. 1). To our knowledge, this is the third time that *P. markhami* has been collected, always parasitizing the branchial chambers of the prawn *M. americanum*.

Remarks.– The characters of the specimens examined (Figs. 2B, 3B, 4C-D, 6A) are consistent with those proposed for *P. markhami* by Román-Contreras (1996), excepting the following: barbula with middle margin slightly sinuated (Fig. 6B); maxilliped with triangular palp but of more rounded outline than illustrated by Román-Contreras (1996, figs. 8–9) and the number of setae on it varied from three to ten (Fig. 6C); first pair of oostegites with posterolateral point wide and rounded but not horn-shaped (Fig. 6D, E), and inner margin with stout and semi-quadrate digitations (Fig. 6E); conspicuous dark brown carina on basis of all pereopods on short side of body (Figs. 3B, 6F); pleopods 1 and 2 with endopod rounded and wider than exopod while in pairs 3–5 both rami are progressively more similar in size and shape posteriorly (Fig. 6F).

*Probopyrus markhami* resembles *P. pacificensis*, but according to Román-Contreras (1996) they can be distinguished by the body length/width ratio, the depth of the notch of the last pleomere, the body pigmentation intensity, the pleopods’ length and the presence/absence of tiny knob-like uropods near the anterior margin of the ventral surface of the last pleomere. Based on the material examined of both species we suggest that the shape of both the palp and digitations of the inner ridge of the first oostegite are characters to distinguish between species.

Román-Contreras (2004) recognized four *Probopyrus* species distributed along the Mexican Pacific: *P. bithynis* Richardson, 1904, *P. markhami*, *P. pacificensis* and *P. pandalicola*. The presence in the Eastern Pacific of the latter two species was treated above. The record of *P. bithynis* from the Mexican Pacific coast seems dubious because it was recorded parasitizing *Macrobrachium olfersii* (Wiegmann) at Tuxtepec River, Oaxaca, a branch of the Papaloapan River (Román-Contreras, 2004) which originates in Oaxaca, near the border with Veracruz, and flows into the Gulf of Mexico. Apparently, this host-parasite association can inhabit regions very far from the coasts, as they have also been recorded in the Tamuín River, San Luis Potosí, 200 km inland from the Gulf of Mexico (Román-Contreras, 2004). Hence, we suggest that *P. markhami* and *P. pacificensis* are the two only *Probopyrus* species distributed along the Pacific coast of Mexico.

Table 1 shows the average fecundity and embryo size recorded in two ovigerous females of *P. markhami*, to our knowledge, these are the first reproductive data for this bopyrid. The fecundity calculated for *P. markhami* is among the highest recorded for any bopyrid (see Cericola & Williams, 2015; Romero-Rodríguez & Álvarez, 2020; 2023) which could be explained by the large size of the ovigerous female examined (~18 mm TL) because in bopyrids fecundity is positively related to the size of ovigerous females (see Cericola & Williams, 2015). Sizes of embryos in stage I (Table 1) are similar to those previously reported for other bopyrid species but epicaridium larval size (Table1) is slightly larger than those of other species (see Romero-Rodríguez and Álvarez, 2020, 2023).

Genus *Schizobopyrina* Markham, 1985

***Schizobopyrina striata*** (Nierstrasz & Brender à Brandis, 1929)

Figs. 1, 2C, 3C, Table 1

*Bopyrina striata* Nierstrasz & Brender à Brandis, 1929: 40–42, figs. 52–53 [type-locality: San Diego Bay, California, USA, parasitizing *Hippolyte californiensis* Holmes].

*Schizobopyrina striata*.– Markham, 1985: 46.– Campos & Campos, 1990: 634–637, figs. 1–2 [Puertecitos, Baja California, Mexico, parasitizing *Thor algicola* Wicksten].–Markham, 1992: Table 1.– Espinoza-Pérez & Hendrickx, 2001: 51.– Brusca et al., 2005: 137.– Román-Contreras, 2008: Table 1.– Espinoza-Pérez & Hendrickx, 2006: 237.– Brusca, 2007: 426, 493 Table 29.3.– Romero-Rodríguez & Martínez-Mayén, 2017: 119.– Hendrickx et al., 2019: Table 7.

Material examined.– 1 ovigerous female of 3.27 mm TL (CNCR-36924) parasitizing 1 *Periclimenes infraespinis* (Rathbun) female of 3.03 mm CL (CNCR-29152); J. Romero det. host; Isla Alcatraz, Bahía de Kino, Sonora, Mexico (28º49'00.4''N 111º58'58''W); 08 March 2007.

Distribution.– To our knowledge this is the third time that *S. striata* has been collected since it was described, and the second record from the Gulf of California (Fig. 1). It has been recorded from San Diego, California, USA; Puertecitos, Baja California (Campos & Campos, 1990) and Isla Alcatráz, Sonora, Mexico (Herein). Although it is distributed in a relatively restricted region this bopyrid species has a wide host selectivity as it occurs in both *H. californiensis* and *T. algicola* (Nierstrasz & Brender à Brandis, 1929; Campos & Campos, 1990) belonging to Alpheoidea and *P. infraespinis* (Herein) to the Palaemonidae.

Remarks.– Excepting the lack of dorsal or ventral pigmentation, the female examined (Figs. 2C, 3C) fits well all other characters previously reported for *S. striata* by Nierstrasz & Brender à Brandis (1929) and Campos & Campos (1990). The female examined was almost detached from the host’s branchial chamber, which could explain the absence of the male and the evident brood mass loss, as it was only carrying 25 embryos in stage I, with sizes (Table 1) similar to those recorded for other bopyrid species (see Romero-Rodríguez & Álvarez, 2020, 2023).

Subfamily Hemiarthrinae Markham, 1972

Genus *Cataphryxus* Shiino, 1936

***Cataphryxus zapoteca*** **sp. nov.**

Figs. 1, 2D, 3D, 7

**Fig. 7:**
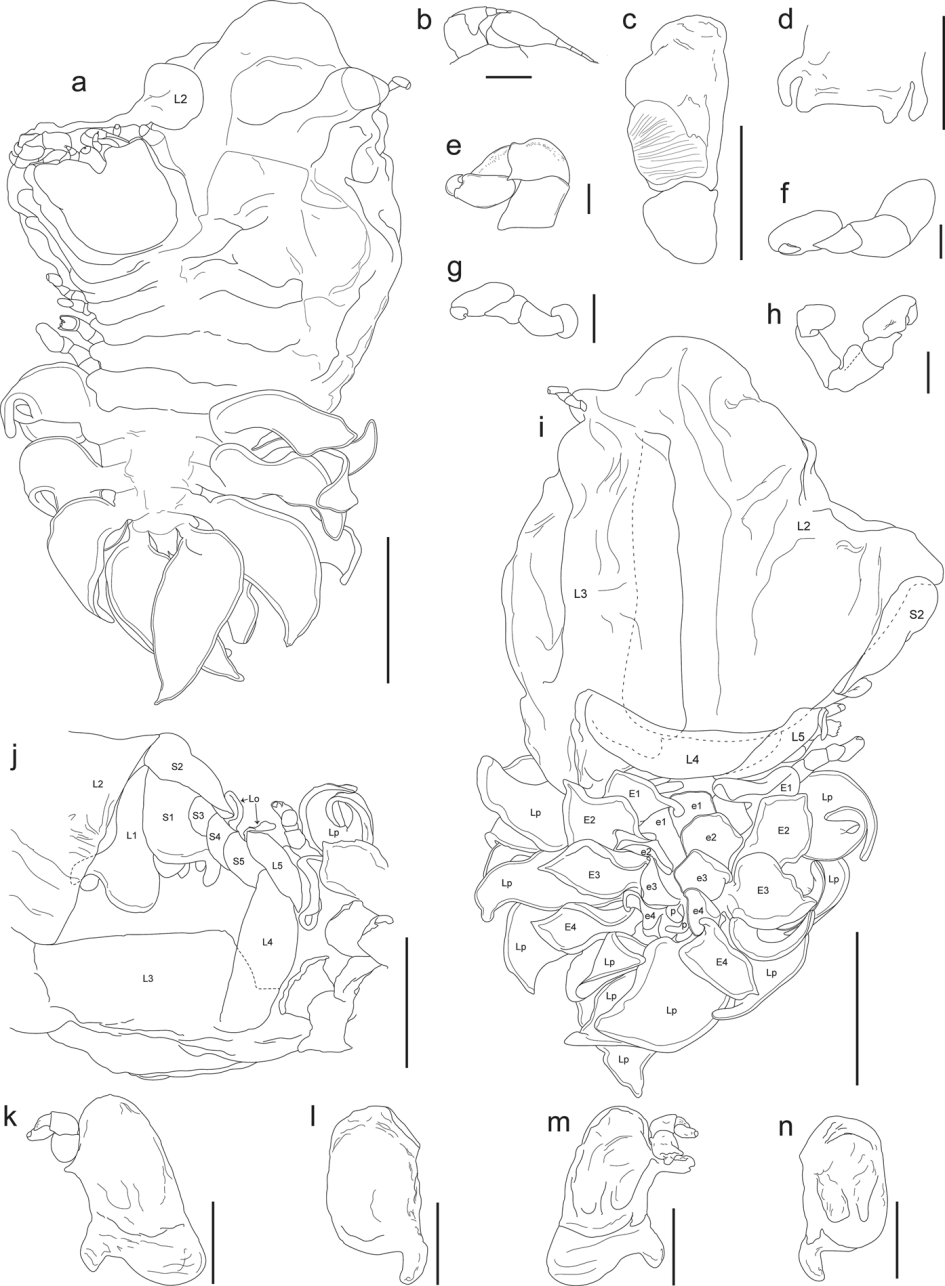
*Cataphryxus zapoteca* **sp. nov.** CNCR-36925. **a.** Adult female holotype, dorsal view. **b.** Antennule and antennae. **c.** Maxilliped. **d.** Barbula. **e.** Pereopod 1, short side. **f.** Pereopod 7, short side. **g.** Pereopod 3, long side. **h.** Pereopod 7, long side. **i.** Adult female holotype, ventral view. **j.** Oostegites detail. **k.** Oostegite 1 with pereopod 1, long side, external view. **l.** Oostegite 1, short side, external view. **m.** Oostegite 1 with pereopod 1, long side, internal view. **n.** Oostegite 1, short side, internal view. Scale bars: **a**, **j** and **i** = 1.0 mm. **c**-**d** and **k**-**n** = 0.5 mm. **b** and **e**-**h** = 0.1 mm. Numbers indicate the corresponding structures. Abbreviations: **E**, exopod; **e**, endopod; **L**, oostegite of long side of body; **Lo**, external lobe; **Lp**, lateral plate; **p**, papilla-like appendage; **S**, oostegite of short side of body

Type host.– *Lysmata galapagensis* Schmitt.

Type locality.– Estacahuite beach, Oaxaca, Southeast Pacific coast of Mexico (15°40'06"N 96°28'53"W).

Type Material.– 1 adult female of 3.52 mm TL (CNCR-36925) on the abdomen of *L. galapagensis* male of 5.87 mm CL (CNCR-31526); Martínez-Guerrero B. det. host; Estacahuite, Oaxaca, Mexico at 20 m. deep; 28 February 2015.

Etymology: The specific name *zapoteca* is to honor to the ancient Mesoamerican civilization that inhabited the current region of Oaxaca, mainly, where the specimen was collected.

Diagnosis.– Female body asymmetrical, right side of body longer than left, all pereomeres distinct on short side of body, fused on long side, 7 pairs of pereopods, marsupium tightly closed, oostegites of long side of body larger than on short side, 5 pleomeres, bilobed lateral plates on pleomeres 1–4, pleomere 5 small with bilobed posterior margin, 4 pairs of biramous pleopods but 3 and 4 each with additional appendage next to endopods, uropods absent. Body white in color, lacking pigmentation and eyes.

Description.– Holotype: Adult female (Figs. 2D, 3D, 7A): body length 3.52 mm, maximal width 2.71 mm at pereomere 3, head length 0.84 mm, head width 1.00 mm, pleon length 1.09 mm, pleon width 2.80 mm. Head square in shape, distinct from first pereomere, posterior margin slightly rounded, anterior margin deeply divided at middle portion, lateral margins extending onto first pereomere, eyes absent (Figs. 2D, 7A). Antennule of 4 segments, basal one largest with rounded margins, distal segment smaller, rounded and bearing 2 or 3 distal setae (Fig. 7B). Antenna of 5 segments, first one short and broad, second one larger and tapered, three last segments very slender, of similar size but faintly tapering distally, last segment bearing 2 or 3 distal setae (Fig. 7B). Maxilliped longer than wide, without palp, surface smooth, anterior segment semi-rectangular in shape with anterior margin rounded, slightly tapered posteriorly; posterior segment triangular in shape with small and blunt spur (Fig. 7C). Barbula with 2 smooth projections on each side; external projection slender, blunt and slightly curved; internal projection wider, blunt and triangular in shape; medial margin nearly straight and smooth (Fig. 7D).

Pereon with all pereomeres on short side of body distinct from mid-dorsal portion to lateral margins; on long side of body, excepting pereomeres 1 and 2, both dorsal and lateral margins not clearly distinct (Figs. 2D, 7A). Pereomeres 1 and 2 with small, flat and rounded dorsolateral bosses. Pereomeres 3 and 4 of long side of body with indistinct square dorsolateral bosses, both pereomeres far apart from each other, pereomere 4 reaching first pleopods (Fig. 7A). Seven pairs of pereopods; first 2 pairs next to head, directed forward and similar in size and form; basis and ischium stout and square in outline, merus and carpus fused, propodus oblong, dactylus short and blunt (Fig. 7E). Pereopods 3–7 on short side of body close to each other (Fig. 2D) and of similar form to first 2 pairs but slightly increasing in size posteriorly (Fig. 7F). On long side of body pereopods 3–7 thinner and differing in form, with bases of 3 and 4 short and rounded, ischium long, merus and carpus fused, propodus oblong and dactylus tiny and blunt, both widely separated from each other and on lateral margin of pereomeres 3 and 4, respectively (Fig. 7G); pereopods 5–7 with bases each as rounded bump, ischium long, merus with lateral carina, carpus semi-square, propodus oblong and dactylus short and blunt (Fig. 7H), all three crowded between posterolateral margin of oostegite 4 and first pair of pleopods of long side of body (Fig. 3D). Marsupium completely closed by oostegites of long side of body (Figs. 3D, 7I), oostegite 2 roughly triangular, covering more than half of marsupium with projecting lobe extending between pereomeres 2 and 3 (Fig. 7A, I); oostegites 3–5 of rectangular shape, decreasing in size posteriorly, oostegites 2–4 with bases of lateral margins fused (Fig. 7I, J). First pair of oostegites with smooth surface, larger on long side of body than short side but similar in shape, anterior segment larger and ovoid, posterior segment short with rounded margins and stout posterolateral point (Fig. 7K, L); inner ridge curved and smooth with thick and triangular lobule on proximal portion (Fig. 7M, N). On short side of body, oostegite 2 ovoid, larger than first one,overlapping oostegites 1, 3, 4; oostegites 3–5 reduced, ovoid in shape and imbricated, with external lobe on oostegites 4 and 5 (Fig. 7J).

Pleon with 5 pleomeres laterally distinct but more or less fused medially, tapering posteriorly (Figs. 2D, 7A). Pleomeres 1–4 with bilobed lateral plates arising from short peduncle, both lobes similar in size, elongated and oval, lateral plates on pleomeres 1 and 2 more recurved than on 3 and 4 (Fig. 7A, I, J). Pleomere 5 small, quadrangular in shape with bilobed posterior margin (Fig. 7A). Pleon nearly covered dorsally by 4 pairs of pleopods. Exopods larger than endopods, foliose with irregular outline and more or less folded; endopods square in shape and irregular outline, pleopods 3 and 4 each with extra papilla-like appendage next to base (Figs. 3D, 7I). Uropods absent.

Male: Unknown.

Remarks.– According to the female’s morphological characters (pereomeres fused on long side of body, 7 pairs of pereopods, 5 pleomeres, of which the first 4 with bilobated lateral plates), the specimen examined belongs to *Cataphryxus* (Shiino, 1936). No male was recorded but males of this genus have the head separated from the first pereomere, pleomeres are completely fused, and lack pleopods and uropods (Shiino, 1936).

Boyko et al. (2023) listed this genus as monotypic, represented only by *Cataphryxus primus* (Shiino, 1934). Females of *C. zapoteca* **sp. nov.** and *C. primus* are quite similar, but can be distinguished by *C. primus* having pereomeres lacking dorsolateral bosses, pleomeres laterally and dorsally distinct, lateral plates of pleomeres 1–4 and pleopods similar in shape, only 4 oostegites on the long side of the body, maxilliped with nearly straight anterior margin, and barbula with external projection curved and hook-like (Shiino, 1934).

*Cataphryxus* species parasitize the abdomen of *Lysmata* shrimps from the Pacific Ocean, *C. primus* on *Lysmata* sp. (referred as *Hippolysmata* sp.) from Yusaki, Seto, Japan (Shiino, 1934) and *C. zapoteca* **n. sp.** on *L. galapagensis* from the southeast Pacific coast of Mexico.

Subfamily Pseudioninae Codreanu, 1967

Genus *Munidion* Hansen, 1897

***Munidion pleuroncodis*** Markham, 1975

**Fig. 8:**
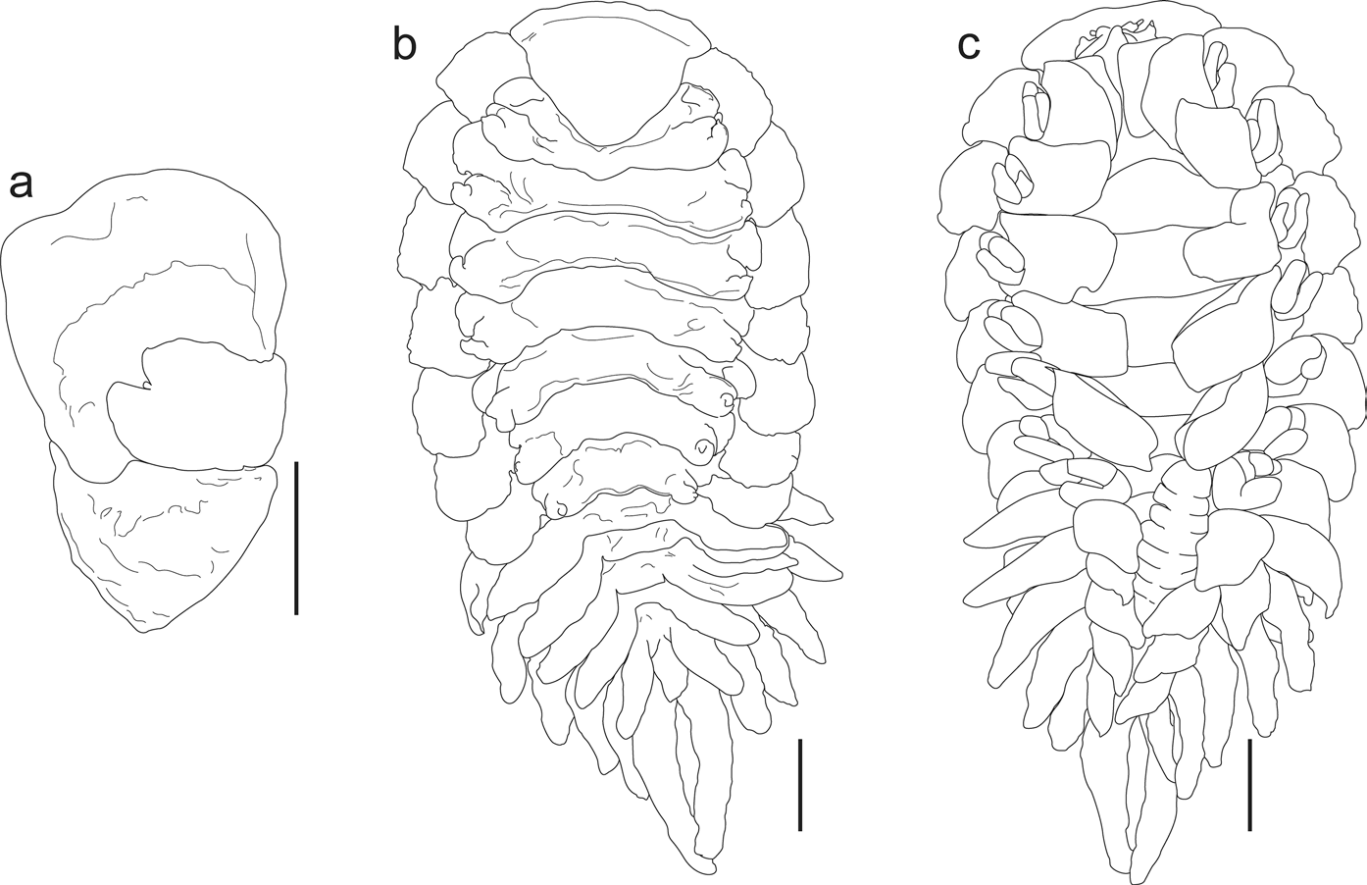
*Munidion pleuroncodis*. **a.** Maxilliped of adult female (CNCR-36926-F). **b.** Juvenile female, dorsal view. **c.** same, ventral view (CNCR-36926-E). Scale bars = 1.0 mm

Figs. 1, 2E, 3E, 4E-F, 8, Table 1

*Munidion pleuroncodis* Markham, 1975: 425–428, 432, 440 (in key), figs. 5–8 [type locality: off Baja California Sur, Mexico, parasitizing *Grimothea planipes* (Stimpson) (cited as *Pleuroncodes planipes*)].– Campos-González & Campoy-Favela, 1987: 39, 46–47 (in key).– Markham, 1992: Table 1.– Brook et al., 1994: Table 1.– Wetzer & Brusca, 1997: 25–27, figs. 1.9, 1.10.– Pardo et al., 1998: 276.– Tsai et al., 1999: Table 2.– Brusca et al., 2007: 512 (in key), fig. 236C.– Román-Contreras, 2008: 93, 94 Table 1.

Material examined.– 9 ovigerous females (13.20 ± 1.04 mm TL), 2 adult females (12.85 ± 0.33 mm TL), 1 juvenile female (10.77 mm TL) and 12 males (2.29 ± 0.24 mm TL) (CNCR-36926) parasitizing 7 females (24.30 ± 2.14 mm CL) and 5 males (28.59 ± 0.93 mm CL) of *Grimothea planipes* (CNCR-9198); J.L. Villalobos det. host; Isla Socorro, Colima, Mexico (18°46'26"N 110°58'18"W); 08 May 1965.

Distribution.– *Munidion pleuroncodis* has been recorded from Monterey, California, USA, to the west coast of the Baja California peninsula, Mexico (Markham, 1975, 1992). Wetzer & Brusca (1997) suggested that it occurred at least to the central part of Mexico, but to our knowledge the record from Isla Socorro, Colima, represents the southern limit of its known distribution (Fig. 1). The species is always found parasitizing the branchial chambers of the galatheid “red crab” *G. planipes*.

Remarks.– The characters of the females (Figs. 2E, 3E) and males (Fig. 4E-F) examined matched well with those proposed for *M. pleuroncodis* by Markham (1975), excepting the following: all females with maxilliped clearly segmented, posterior segment triangular in outline, provided with long and acute spur (Fig. 8A); one female with the inner margin of the first oostegite barely sinuated. Males each with a midventral tubercle on first pleomere (Fig. 4F). A juvenile female (Fig. 8B, C) showed the marsupium open as the oostegites were not fully developed and both the inner ridge of first oostegite and the middle margin of the barbula are barely sinuated.

To our knowledge, there are no previous reproductive data for this species. The average fecundity of *M. pleuroncodis* was the second highest of all species treated in this study but the sizes and volume of embryos are similar to the other species reported here (Table 1) and to other bopyrids (Romero-Rodríguez & Álvarez, 2020, 2023). Overall, the oostegites of ovigerous females were tightly overlapped but fecundity was quite variable, even in females of similar sizes, which could be attributed to the sampling stress produced during the collection of its host.

Genus *Progebiophilus* Codreanu & Codreanu, 1963

***Progebiophilus bruscai*** Salazar-Vallejo & Leija-Tristán, 1990

**Fig. 9:**
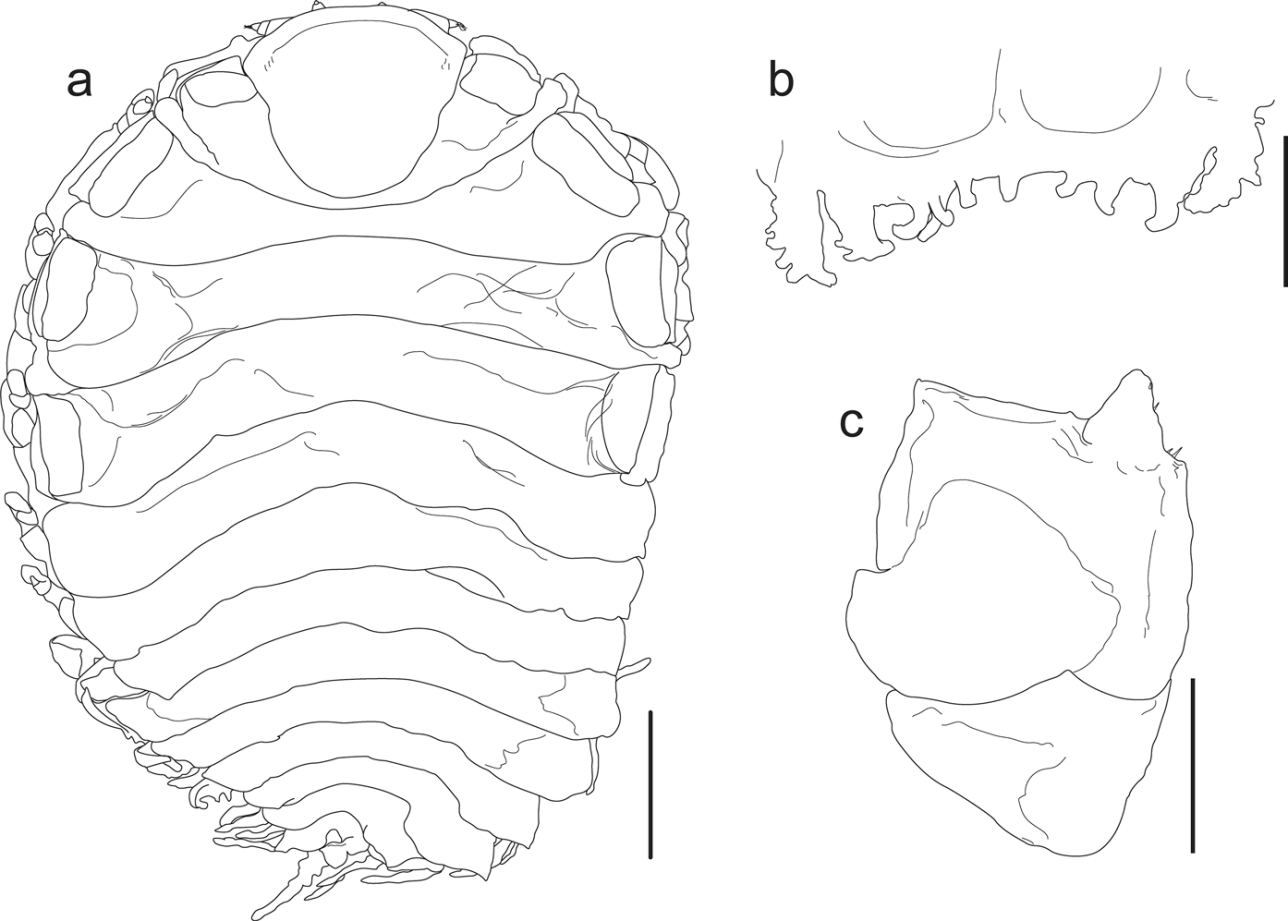
*Progebiophilus bruscai* CNCR-36927. **a.** Ovigerous female, dorsal view. **b.** Barbula. **c.** Maxilliped. Scale bars: **a** = 1.0 mm. **b** and **c** = 0.5 mm

Figs. 1, 2F, 3F, 4G-H, 9, Table 1

*Aporobopyrus* sp.– Leija-Tristan & Salazar-Vallejo, 1987: 179. [de la Paz Bay, Baja California Sur, Mexico, parasitizing *Upogebia dawsoni* Williams]

*Pseudione* sp.– Campos & Campos, 1989a: 33, Table 2 [Tortugas Bay, Baja California Sur, Mexico, parasitizing *Upogebia macginitieorum* Williams].– Campos & Campos, 1989b: 177 [Todos Santos Bay, Baja California Sur, Mexico, same host].

*Progebiophilus bruscai* Salazar-Vallejo & Leija-Tristán, 1990: 424–429, figs. 2, 3, Table 1 [type locality: de la Paz Bay, Baja California Sur, Mexico, parasitizing *U. dawsoni*]

*Progebiophilus bruscai*.– Leija-Tristán & Salazar-Vallejo, 1991: 1-4, fig. 1, Tables 1, 2.– Campos et al. 1992: 753, 756, 757.– Markham, 1992: Table 1.– Kazmi & Bourdon, 1997: 62.– Campos & Campos, 1998: 288–293, figs. 1, 2, Table 1 [Baja California and Baja California Sur, Mexico, parasitizing *U. dawsoni* and *U. macginitieorum*].– Trilles, 1999: 326.– Espinoza-Pérez & Hendrickx, 2001: 51.– Markham, 2001: 198, 200.– Brusca et al., 2005: 137.– Markham, 2005: 85, 86, 90 [Baja California and Baja California Sur, Mexico, parasitizing *U. dawsoni* and *Pomatogebia rugosa* (Lockington) (cited as *Upogebia rugosa*)].– Espinoza-Pérez & Hendrickx, 2006: 237.– Román-Contreras, 2008: Table 1.–Smith et al., 2008: 231.– An et al., 2009: Table 1.– Campos et al., 2009: 1255, 1257, Table 1.– Williams & An, 2009: 121.– Dumbauld et al., 2011: 337.– Boyko et al., 2017: 268, 269.– Hendrickx et al., 2019: Table 7.– Romero-Rodríguez & Álvarez, 2019: 100.– Bortolini et al., 2021: Table 2. Aguilar-Perera, 2022: 114, Table 1.

Material examined.– 1 ovigerous female of 6.53 mm TL (CNCR-36927) on 1 *Upogebia galapagensis* Williams female of 9.50 mm CL (CNCR-6761); J.C. Nates det. host; Isla San José, near to Cocinas estuary inlet, Baja California Sur, Mexico (24°53'14"N 110°34'24"W); E. Lira and M.D. Valle colls.; 04 November 1986.

Distribution.– *Progebiophilus bruscai* parasitizes the branchial chamber of ghost shrimps of the genus *Upogebia* (*U. dawsoni*, *U. macginitieorum* and *U. spinigera* (Smith)) and *Pomatogebia* (*P. rugosa*) that inhabit both coasts of the Baja California peninsula, Mexico (Fig. 1), and Santa Julia, Nicaragua (Boyko et al., 2017). Our record is within the known distribution range of this parasite but *U. galapagensis* is reported for the first time as host of *P. bruscai*.

Remarks.– The female (Figs. 2F, 3F) and male (Fig. 4G-H) examined resemble *P. bruscai* in all details, except the following: female with pereomeres 1–4 bearing reduced and rectangular coxal plates as well as flattened and rectangular dorsolateral bosses (Fig. 9A); barbula with two thin lateral projections of similar size on each side and with the right middle margin more digitate than the left (Fig. 9B); maxillipeds with anterior segment quadrangular in outline, posterior one triangular with acute spur and triangular palp bearing scant thin setae (Fig. 9C). The male has mid-ventral tubercles on pereomeres 4–7 and lacks terminal setae on the uropods (Fig. 4G-H). No previous reproduction data for *P. bruscai* is available but fecundity and embryo size recorded from the female examined (Table 1) are similar to those reported for other bopyrids of comparable size (see Cericola & Williams, 2015; Romero-Rodríguez & Álvarez, 2020, 2023).

## Discussion

The epicaridean (Bopyroidea and Cryptoniscoidea) biodiversity reports from the Pacific coast of Mexico have been summarized by Campos & Campos (1990a), Salazar-Vallejo & Leija-Tristán (1990) and Román-Contreras (2008), and have also been included in compilations on the diversity of crustaceans that inhabit this region (Espinosa-Pérez & Hendrickx, 2001; Aguilar-Perera, 2022; García-Madrigal et al., 2022). However, further data including prevalence, annual abundance variability or host-parasite size relationship have been reported for only two of six bopyrid species examined here: *Probopyrus pacificensis* (see Guzmán & Román-Contreras, 1983; Campos & Campos, 1989a; Vargas-Ceballos et al., 2016) and *Progebiophilus bruscai* (see Leija-Tristán & Salazar-Vallejo, 1991). To our knowledge, the first records on the reproductive biology for all species examined, excepting *Cataphryxus zapoteca* **sp. nov.**, are here reported.

Overall, ovigerous females of the larger species, *P. markhami* and *M. pleuroncodis*, carried a greater number of embryos (Table 1) which is consistent with the assumption that fecundity in bopyrids is positively related to female size (Beck, 1980; McDermott, 1991, 2002; Romero-Rodríguez & Román-Contreras, 2013). Likewise, it is important to highlight that embryo sizes data for the 5 species examined were comparable to those reported for other bopyrids (Cericola & Williams, 2015; Romero-Rodríguez & Álvarez, 2020, 2023), and provides an initial insight on reproductive traits of these bopyrid species, as the information comes from samples not systematically collected for isopod parasites; thus, further studies are needed in order to get a better understanding of the reproductive behavior of this parasitic group in the Eastern Pacific.

The bopyrid biodiversity for the Mexican Pacific remains poorly known, both taxonomically and biologically and, although *C*. *zapoteca* **sp. nov.** represents a new record, the number of species of these parasites recognized in this region remains at 19 due to the removal of *P. pandalicola* whose distribution range is restricted to the Atlantic coast. Most bopyrid species are distributed in the Gulf of California area and only five species (*Aporobopyrus trilobatus* (Nierstrasz & Brender à Brandis, 1925), *P. markhami*, *P. pacificensis*, *P. ornata*, and *C*. *zapoteca* **sp. nov.**) have been recorded in the Mexican South Pacific region (Fig. 1). The distribution of bopyrids coincides with the general crustacean diversity observed throughout the Mexican Pacific, which is higher in the Gulf of California area, south to Cabo Corrientes, Jalisco, and decreases in the South Pacific (Hendrickx, 1993); this could be an artifact of the reduced number of samplings done in this region (García-Madrigal et al., 2012). Recently, efforts have been made to improve our knowledge of the crustacean diversity in the Mexican South Pacific (García-Madrigal et al., 2022), which could also improve the data on the symbionts, including bopyrid isopods, associated with these crustaceans.

## Data Availability

Type material, and all specimens examined, are deposited in the Colección Nacional de Crustáceos (CNCR), Instituto de Biología, Universidad Nacional Autónoma de México (see text for details) and are available for study. Collections data are available in CNCR registers.
